# The relation between maximal voluntary force in *m. palmaris longus* and the temporal and spatial summation of muscle fiber recruitment in human subjects

**DOI:** 10.14814/phy2.13580

**Published:** 2018-01-15

**Authors:** Cécyl G. Claudel, Waqas Ahmed, Vibeke S. Elbrønd, Adrian P. Harrison, Else Marie Bartels

**Affiliations:** ^1^ IVH Faculty of Health & Medical Sciences Copenhagen University Copenhagen Denmark; ^2^ The Parker Institute Copenhagen University Hospital Bispebjerg & Frederiksberg Copenhagen Denmark

**Keywords:** Acoustic myography, grip strength, hand strength, muscle contraction, sonography

## Abstract

This study aimed at looking at the frequency (T‐score) and the amplitude (S‐score) of fiber use during contraction of a forearm muscle, *m. palmaris longus*, as measured by acoustic myography (AMG). An additional aim was to relate the T‐ and S‐scores to the recorded force obtained from a hand dynamometer. The hypothesis being that temporal and spatial summation of muscle fiber contraction in a given muscle during a given movement, can together describe a given obtained force. Force measurements were carried out on 12 healthy human subjects aged 19–68 years (6 men & 6 women), while their *m. palmaris longus* contractile function was measured using an acoustic myography CURO device. Force production was varied from 90 to 10% of assessed maximal voluntary force (MVF), and also monitored over a 1 min period of 50% MVF. Linear regression analysis was applied to relate force to spatial and temporal summation. Muscle strength was sustained by changing the frequency and/or the number of active fibere at any given point in time. Force production, whilst stronger for men than women, was regulated in a similar fashion for both sexes and was closely correlated with the AMG T‐ and S‐scores. It is concluded that AMG is a noninvasive method which can be readily applied to accurately describe how a subject uses a given muscle during any given movement. These findings have relevance when considering training strategies in subjects with muscle trauma or disease, in the elderly, or for both amateur and top professional athletes.

## Introduction

Whilst it is well known that the ability to hold and maintain a firm grip on something is continuously regulated by the central nervous system (CNS) (Johannson and Westling 1984; Edin et al. 1992; Burnely and Jones 2016), accurate correlations of muscle force with the recorded signal from contracting fibers remain less well documented. When producing a given muscle force, recruitment of the fibers in a muscle may happen by either recruiting many fibers at any given time or by using fewer fibers, but firing them at a higher frequency (Carlson and Wilkie 1974; Contessa et al. 2016).

Surface electromyography (sEMG) recordings are frequently used to assess muscle function in a noninvasive way. Indeed, the technique has more or less become a gold standard (De Luca 1997; Hermens et al. 1999). However, sEMG is subject to such complex issues as sampling rate, noise and interference, and such specific issues as causative, intermediate and deterministic factors that can and do affect the recorded signal, making it difficult and complex to interpret – see the SENIAM report (Hermens et al. 1999). The use of sEMG appears to be further confounded by older and more recent investigations into muscle contractility and force production. These confirm the existence of other confounding issues in terms of signal recording and accurate interpretation, including motor neuron interference, as well as modulating processes ranging from development, metabolism, blood flow and contractile function in muscles (Bevington et al. 1986; Reid 2001; Harrison et al. 2006; Harrison 2017). It is for these very reasons that an alternative to sEMG, one that accurately records muscle fiber contractions alone, has been sought in recent years. Indeed, just such a promising alternative is to be found in Acoustic Myography (AMG) (Stokes and Blythe 2001; Harrison et al. 2013).

The principle aim of this study was to look at the frequency and amplitude of muscle fiber use during voluntary contractions of a forearm muscle, *m. palmaris longus,* expressed by the T‐ and the S‐score (ESTi™‐score; MyoDynamik ApS, Frederiksberg, Denmark) measured by the AMG technique. An additional aim was to relate these parameters to recorded muscle force obtained, using a professional hand dynamometer.

The hypothesis we wished to test in this study was that temporal and spatial summation of fiber contraction together, describe a given obtained force in a skeletal muscle during voluntary contraction.

The scope behind this study was that AMG can be applied when wishing to get a picture of efficiency of muscle use and development of muscle fatigue during a defined movement. This may help when assessing the functionality of elderly subjects and subjects with musculoskeletal diseases, as well as define and guide the training of amateur and top performance athletes. In order to better assess the universal and fundamental importance and accuracy of spatial and temporal summation in voluntary force production, as assessed using AMG, a broad age range of subjects was selected.

## Materials and Methods

### Subjects

12 healthy human subjects, 6 men aged 19–53 (Mean ± SD; 35.3 ± 13.7) and 6 women aged 21–68 (Mean ± SD; 45.2 ± 16.8), were measured using AMG for muscle function of *m. palmaris longus* during grip strength measurements performed when voluntarily performing gripping activities with a dynamometer. The number of subjects measured using their dominant right hand was 12, and of these 6 were female. Owing to the fact that approximately 14% of the population does not have *m. palmaris longus*, we performed a simple test to ensure that our subjects possessed this muscle. The subjects were asked to place their thumb and little finger gently together, before being asked to bend their wrist to reduce the angle of the hand to the forearm. For those subjects with *m. palmaris longus*, the *palmaris longus* tendon should appear raised and obvious at the midpoint of the wrist, as was the case for the subjects recruited in this trial.

### Ethics

The subjects were informed about the study and were able to see the measuring setup and ask questions in a private setting before consenting to participate. There was no ethical issue in this study, since all the subjects were healthy and able to understand the instructions given. Moreover, the measuring equipment used complied with both CE and FCC regulations and was noninvasive in its nature.

The study was carried out according to the Helsinki Declaration (https://www.wma.net/policies-post/wma-declaration-of-helsinki-ethical-principles-for-medical-research-involving-human-subjects/).

### Strength measurements

Strength measurements were carried out using a hydraulic hand dynamometer, SH5001 (AlphaSport, Underwood, Australia).

### Acoustic myography recordings

The AMG technique is a noninvasive and pain‐free means of recording muscle contractions transdermally (Harrison et al. 2013; Harrison 2017).

AMG was recorded with a CURO unit and 20 mm CURO sensors (MyoDynamik ApS, Frederiksberg, Denmark), showing an ESTi score and real‐time recordings on an iPad Air (Apple Inc, Cupertino, CA, USA). The sensors had a frequency recording range of 0.5–20 ± 0.5 kHz, and data was stored on the CURO unit until being further processed using the CURO Clinic data recording system. The CURO sensor was coated with acoustic gel (MyoDynamik ApS, Frederiksberg C, Denmark) before placement on the muscle body at the skin level. The AMG parameters determined were the two individual ESTi‐Score components of fiber recruitment Spatial (S‐score) and Temporal (T‐score) (Harrison 2017). The sensor was held in place over the muscle body for the muscle of interest, using a self‐adhesive bandage (Co‐Plus LF; BSN medical GmbH, Hamburg, Germany).

For further details regarding the ESTi‐Score and signal analysis see (Harrison 2017), In brief, the “i” in the ESTi‐score stands for integrated, such that an ESTi‐score is the integrated mean of the E, the S and the T – scores for a muscle. It is typically used to assess muscle function very quickly, for example, if both ESTi left and right are more or less equal, then there is no or minimal imbalance in terms of force production in the subject. The E‐score represents efficiency, strictly the time a muscle is active compared to inactive, thus a low E‐score indicates a muscle that is active most of the time. The S‐score represents spatial summation, that is to say the way the CNS generates muscle force by recruiting more motor units. This score is inverted for ease of use, so a high S‐score means few motor units active and a low amplitude, and a low S‐score means the opposite, many active motor units and a large amplitude. The other way the CNS generates muscle force is through temporal summation (T‐score), where the firing rate of active motor units is increased. Here the active motor units are fired at a faster rate and as a result they produce more force (effectively they have less time to fully relax). This score is also inverted for ease of use, so a high T‐score means a low firing frequency, and a low T‐score means the opposite. In this way, the CNS can determine the amount of force produced by a muscle through determining the number of active motor units and hence active fibers, as well as the rate at which they are fired. The third factor that is included in the ESTi‐score is the E parameter, that is to say efficiency or synchronization. With training, the CNS becomes more efficient at activating fibers at the same time, reducing the braking/resistance effect of counterbalance (antagonist) muscles. Often in a poorly trained individual, an antagonist muscle is not fully relaxed during the contraction of an agonist muscle, forcing the active muscle to work harder, but with training the CNS becomes more efficient at relaxing antagonist muscles at exactly the point of muscle contraction in an agonist. In summary, therefore, an ESTi ‐score is an overview of the efficiency of contraction, the number of active fibers and the firing rate.

A specially designed parallel CURO sensor was used to enable simultaneous transfer of data to the CURO system, as well as to a PowerLab 26T (LTS) A/D Converter (AD Instruments, Hastings, UK) running version 7.0 of LabChart Software (AD Instruments, Hastings, UK). This parallel sensor had a BNC cable attached so that the signal recorded by the sensor was not only sent to the CURO directly, but the raw AMG signal was also simultaneously sent to an A/D Converter. The CURO signal was amplified (6 dB), converted from an analog to a digital signal and subsequently recorded without any band pass filtering. Data were sampled on the A/D Converter at a rate of 1 KHz and with a 50 Hz notch filter. The data collection took place in a laboratory setting.

### Experimental design

The subject was asked to sit in a chair with their arm (preferably the one used for writing) resting on a table at a comfortable height whilst holding the dynamometer as instructed by the manufacturer. Maximal force production (MVF) was then determined. This MVF of the subjects was measured during a period of encouragement, using a certified dynamometer The CURO sensor was fixed in position for AMG measurements on *m. palmaris longus belly*. It has been reported that a change in position of the AMG sensor on the body of an active muscle, results in a comparable signal with regard to the ESTi‐Score, unlike the sEMG technique (Harrison 2017). Force production was varied from 90 to 10%, with decremental 10% steps (30 sec of force/30 sec of rest), of the previously assessed maximal force. Following this sequence, the subject was asked to rest for 10 min. The subjects could follow their level of voluntary force production on the dynamometer gauge, thereby ensuring that the force produced was kept at the required level throughout the 30 sec period of force production. The handheld hydraulic dynamometer was fitted with a gauge that showed force production in real time. The subjects could freely see and follow their force production during the experiment.

Muscle fatigue was then recorded by asking the subjects to try to maintain a constant grip strength of 50% of maximal strength for 1 min, striving to stay as stable as possible throughout the test. Encouragement was given to try to overcome the issues ‐of discomfort and boredom associated with repeated force production using a hand held dynamometer, issues which might otherwise have led to a false decline in force production. The subject subsequently relaxed and the AMG sensor was removed.

**Figure 1:**
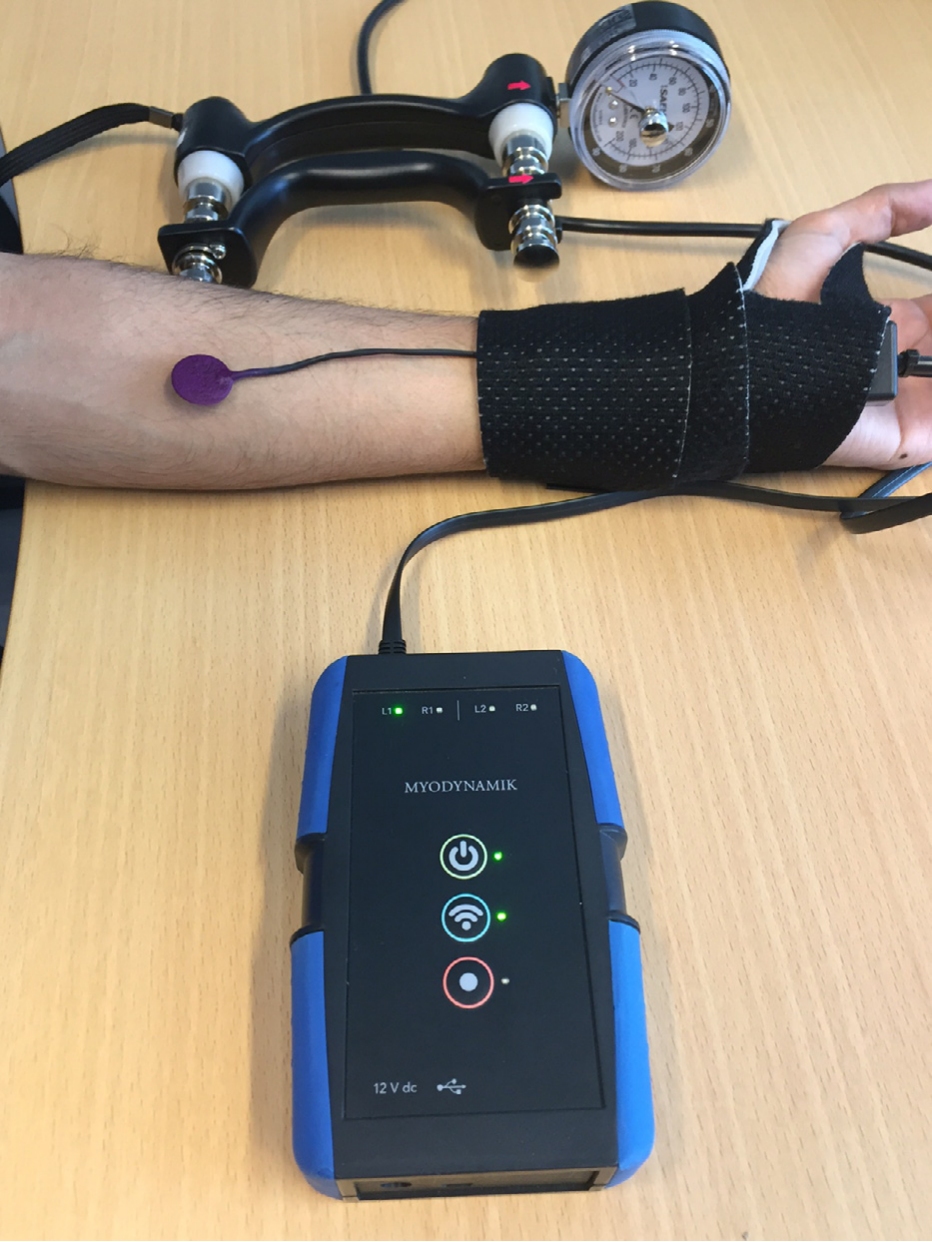
A photograph showing the placement of the AMG sensor on the muscle of interest. Note that a prior test was performed to ascertain whether the subject possessed a *m. palmaris longus*. This placement procedure was adopted for all the measurements. The CURO sensor cable was held loosely in place at the wrist using a reuseable bandage. In the foreground is a CURO unit, with a sensor attached. In the background is the hand hydraulic dynamometer used in this study.

**Figure 2:**
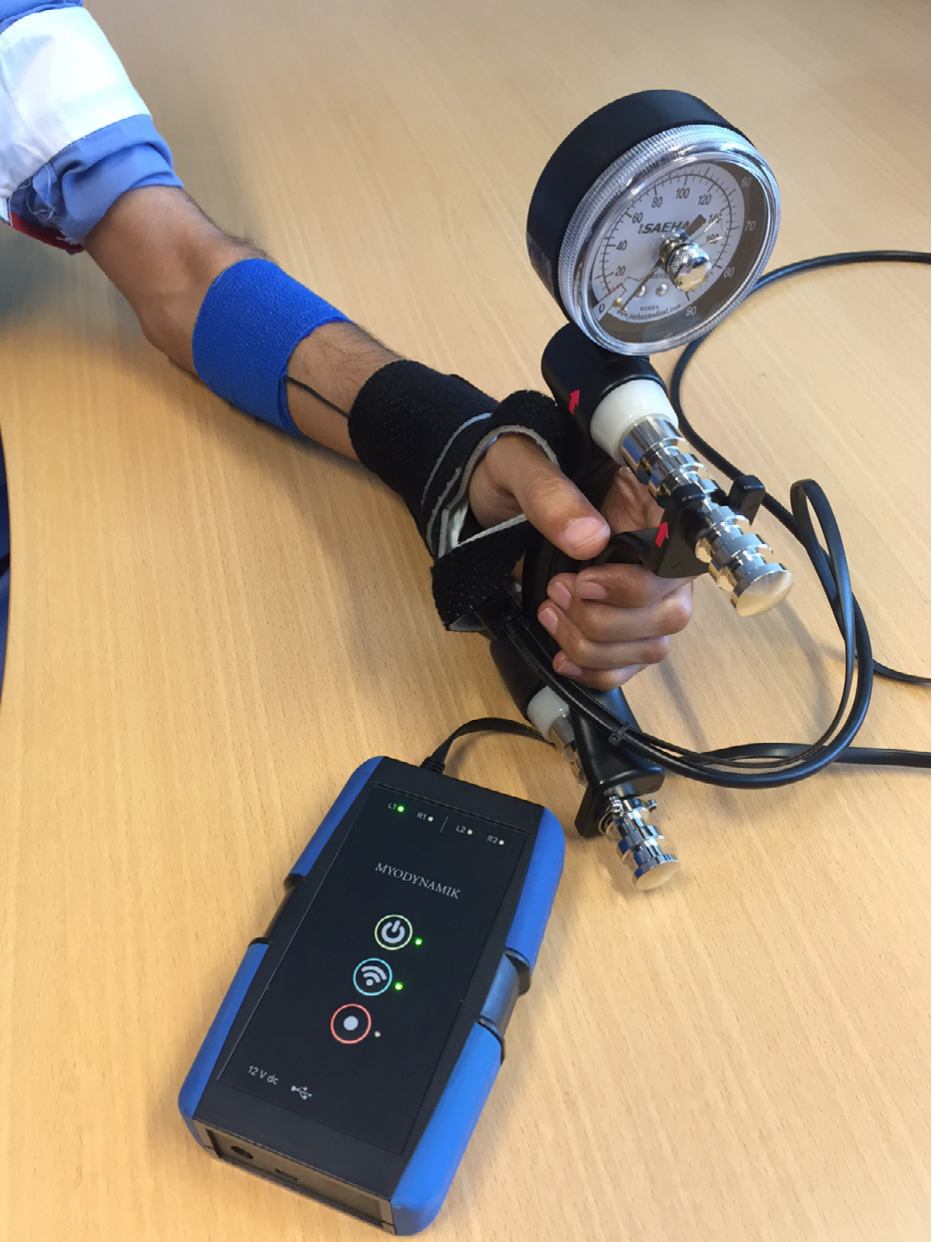
A photograph showing the CURO sensor taped into place on *m. palmaris longus*, and the subject holding the hydraulic dynamometer ready for AMG signal recording at either maximal, decreasing or 50% MVF.

Each recorded time period (90–10% MVF – 30 sec, and 50% MVF – 60 sec) was analyzed in its entirety for both S‐ and T‐scores. Linear regression analyses were applied to relate force production to spatial and temporal summation parameters, as determined using Chart software (v5.5.6; Cycle Variables Frequency & Average Peak‐to‐Peak, AD Instruments, Hastings, UK) of muscle fibers in the involved muscle (Figs. 1 and 2).

### Acoustic myography analysis

When muscle fibers contract, they generate vibrations producing pressure waves within the muscle itself and the surrounding tissues. These waves can be recorded at the level of the skin above the muscle of interest. Thus, AMG should be seen as a transdermal means of recording the produced pressure waves of active muscles.

The CURO system used in this study (MyoDynamik ApS, Frederiksberg C, Denmark) has been designed to perform a signal analysis, which has purposefully been kept as simple and as pure as possible. Only two of the three recorded parameters were subsequently analyzed, the number of active fibers recruited (spatial summation; S‐score), and the frequency with which those active fibers are contracted (temporal summation; T‐score).

The AMG data in this study were analyzed in terms of their individual S and T parameters for each subject (Harrison 2017). The S‐score was determined as the signal amplitude in relation to a full 6 dB signal (0.1 mV), whilst the T‐score was determined as the frequency in relation to a Max T value (120 Hz). Each score is expressed on a scale of 10–0, where 10 is most optimal and 0 is least optimal for each parameter – e.g., a S‐score of 9 represents an AMG signal with a very small amplitude, whilst a T‐score of 2 represents a relatively high firing frequency.

### Statistical analysis

Data were initially tested for a normal distribution and equal variance and then subsequently analyzed, using linear regression for the group as a whole. Differences between means were tested for statistical significance, using GraphPad InStat 3 for Mac (Version 3.0b, 2003; Graph‐ Pad Inc., La Jolla, CA), and then subsequently analyzed, using a regression test. Differences between means with a *P* value >0.05 were considered nonsignificant. Values are presented as the mean ± the standard deviation of the mean.

## Results

### Grip force

**Figure 3:**
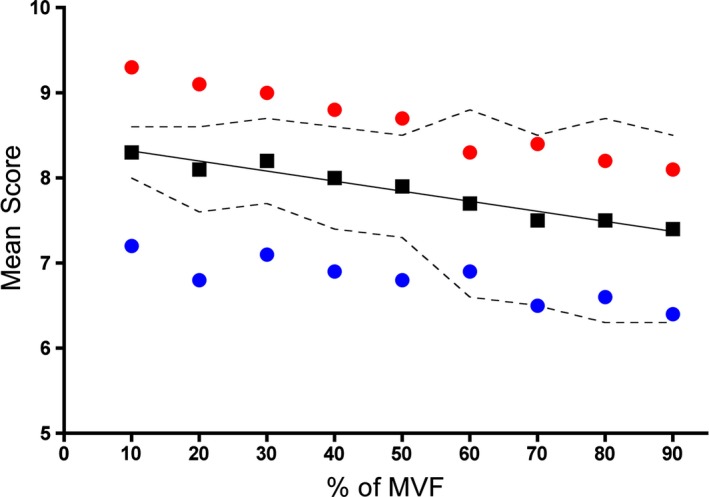
Decline in mean S‐T‐score (black squares) alongside the mean S‐score (red circles) and T‐score (blue circles) values for *m. palmaris longus* with increasing grip force from 10 to 90% of MVF. Values are presented as mean ± SD (dotted lines). The mean of a total number of 12 subjects are presented. The mean MVF was measured to be 380 N.

The mean grip force attained by the 12 subjects in this study was 380 ± 138 N and the maximum voluntary force (MVF) achieved was 580 N, whilst the minimum force was 180 N. The data presented in Figure 3 are for the preferred arm of all 12 subjects.

The mean 50% MVF values for the group was 189 ± 68 N, but it should be noted that the women had a lower mean 50% MVF of 135 ± 29 N, whilst the men had a higher mean 50% MVF of 242 ± 50 N, which was significantly different from that of the women (*P* = 0.001).

### AMG scores in relation to force production

When looking at the mean of the T‐ and S‐scores at each force measurement (between 90 and 10% of maximal grip strength) (Fig. 3), a significant linear relation between this average score and MVF% is seen for all subjects (*r* ^2^ 0.95; *P* < 0.0001), showing a decreasing overall, as well as individual score, with increasing voluntary force production. The data presented in Figure 3 are for the preferred arm of all 12 subjects.

### AMG scores during sustained 50% MVF contraction

**Figure 4:**
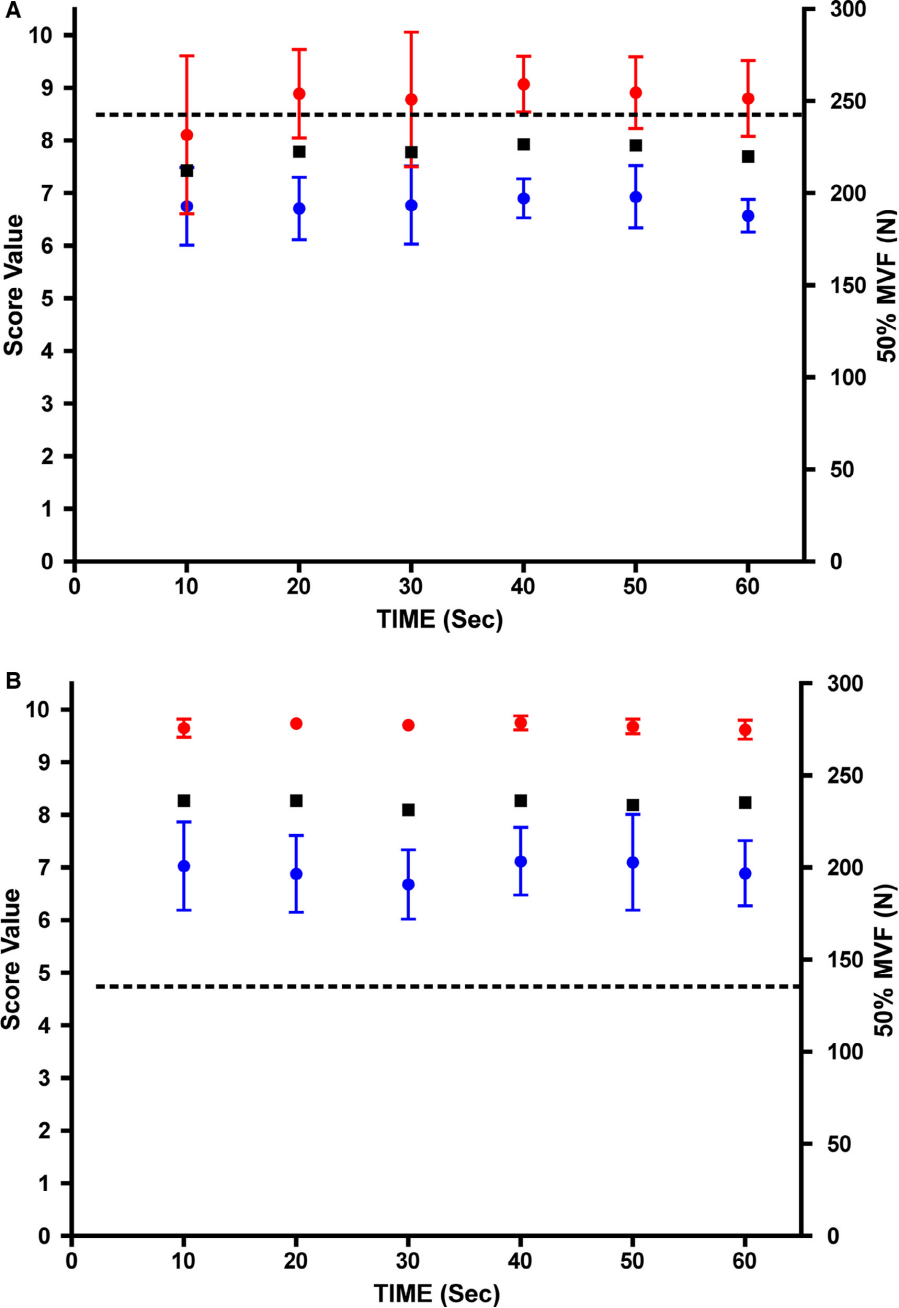
(A) The mean S‐score (red circles) and T‐score (blue circles) for *m. palmaris longus* at mean 50% of MVF (dotted line) for men in this study. In addition, the mean S‐T‐score (black squares) is also plotted. Values are presented as the mean ± SD. *N* = 6 subjects. (B)The Mean S‐score (red circles) and T‐score (blue circles) for *m. palmaris longus* at mean 50% of MVF (dotted line) for women in this study. In addition, the mean S‐T‐score (black squares) is also plotted. Values are presented as the mean ± SD. *N* = 6 subjects.

Looking at the AMG scores for *m. palmaris longus*, maintenance of a stable 50% MVF by the subjects for a period of approximately 1 min resulted in relatively stable T‐ and S‐scores as well as the mean S‐T‐score (Fig. 4). The stability of the AMG scores correlates well with the stable force production over the 1 min of contraction.

## Discussion

This is the first study that has examined voluntary muscle force production as assessed using AMG, and related the voluntary muscle force to the fiber recruitment components of temporal and spatial summation.

It was hypothesized that temporal and spatial summation of voluntary muscle fiber contraction in a given muscle during a given movement would accurately describe the force obtained. With AMG we are able to distinguish between the two ways the CNS controls muscle fiber recruitment to attain voluntary muscle force (Harrison et al. 2013; Harrison 2017). Simultaneous measurement of the AMG signal and the grip strength in *m. palmaris longus* revealed that the hypothesis was confirmed. Our data demonstrate that the mean of the two parameters describing temporal and spatial summation of contracting muscle fibers, T‐ and S‐scores, respectively, precisely relate to the force produced during a voluntary contraction over the range of 90 to 10% of MVF (see Fig. 3). The relatively high T‐score of 7.2 at 10% MVF is equivalent to a firing frequency of 34 Hz, whilst the lower T‐score of 6.4 at 90% MVF is equivalent to a firing frequency of 43 Hz.

It should be noted that at a low level of MVF, the SD for the mean S‐T‐score is rather small, but as the MVF% increases, there is a wider scatter in the SD, which increases with increasing MVF%. It was noted that some subjects maintained a stable S‐score (signal amplitude – number of active fibers), whilst increasing temporal summation. Other subjects preferred to increase spatial summation whilst maintaining a stable level of temporal summation. These coping strategies of the CNS are in accordance with Contessa et al. (2016), who describe the three strategies used; (1) to increase the firing rate of a single motor unit, (2) recruitment of new motor units, and (3) a decrease in motor unit threshold.

The strong correlation between the mean S‐T‐score and MVF% reveals the precision of the AMG technique in terms of accurately detecting muscle fiber contractions as well as its simplicity, comprising the two parameters used by the CNS to generate force in any given muscle.

Subsequently, 50% of MVF was measured for both genders, and plotted to reveal the S‐ and T‐scores for *m. palmaris longus* as well as the mean S‐T‐score (Fig. 4). It can be clearly seen (Fig. 4A) that a stable S‐ and T‐score was maintained over a period of 1 min of voluntary 50% maximal force production, which for men was 242 N. The same result was found for women in this study (Fig. 4B), where an equally stable S‐ and T‐score was found throughout the 1 min period of voluntary 50% maximal force production. Of considerable interest though, is the fact that the mean 50% MVF for women was only 135 N, some 55% of the 50% MVF for men (*P* = 0.001). The AMG finding suggests that the underlying principle of force production in a muscle for men and women is the same. The fact that both the men and women have a very similar T‐score (active fiber firing rate) but clearly differ in their S‐score is rather novel. The mean firing frequency (temporal summation – T‐score) in this study was 43 Hz for the women and 45 Hz for the men throughout the duration of the 1 min period of 50% MVF. What is noticeable though is that the women have a higher S‐score than the men (women = 9.7 S‐score and men = 8.8 S‐score). The higher S‐score for women represents either fewer active fibers, or smaller muscle fibers, or a combination of both of these factors. It is not possible from this study to discern which of these factors results in the underlying weaker 50% MVF for the women compared to the men. However, this is an interesting observation and one that requires further investigation. In studies of resistance training, the difference between men and women are found to be a larger absolute mean muscle fiber area in men compared to women, which may partly come from more type II fibers in men, while the area of type I fibers remains the same for the two sexes (Always et al. 1989, 1992). The much higher MVF in men compared to women is in our study also partly accentuated by men having a higher percentage of muscle mass in the upper body compared to women (Sadbakk et al. 2017). Moreover, when looking at muscle level, the strength is not different between the sexes when normalized to fat‐free body mass or to actual muscle mass (Frontera et al. 1991, Sadbakk et al. 2017).

The 50% MVF protocol adopted in this study did not reveal any clear signs of fatigue, as muscle force was well maintained over the 1 min period of sustained contraction and, likewise, so were the S‐ and T‐scores. It is expected, however, that muscle fatigue, which is dependent on temporal and spatial summation of a muscle, can be revealed using this approach. The present findings confirm those made by Mettler and Griffin (2016) that the neuromuscular system has the ability to sustain the rate of motor unit firing, and that this helps to sustain the submaximal MVF for a longer period, thereby delaying the onset of fatigue. We conclude that this study goes a long way to confirm what was proposed by Carlson and Wilkie (1974), and that it is now time to reconsider the use of this method with its latest improvements.

Our results further confirm that AMG as a method can be applied to describe how a subject uses a given muscle during a given movement, and to assess development of muscle function. With the method being quick and easy to apply, and being noninvasive, it has a wide application in the training field. Furthermore, AMG measurements, unlike the sEMG signal, are quick and easy to analyze, and do not require any correctional data manipulations (e.g., Root Mean Square (RMS) calculations). The method is therefore a recommendable change from sEMG. With its wider applicability, it may be used when considering training strategies in subjects with muscle trauma or disease, when assessing functionality in the elderly, in an athlete setting or indeed as part of a teaching program for physiology using, for example, the PowerLab platform or something similar, which enables real‐time assessment of muscle force production.

## Conflicts of Interests

APH is currently trying to commercialize the AMG recording system and is establishing a company to cover the costs of future development.
